# Spatial Variation in Population Structure and Its Relation to Movement and the Potential for Dispersal in a Model Intertidal Invertebrate

**DOI:** 10.1371/journal.pone.0069091

**Published:** 2013-07-12

**Authors:** Trevor T. Bringloe, David Drolet, Myriam A. Barbeau, Mark R. Forbes, Travis G. Gerwing

**Affiliations:** 1 Department of Biology, University of New Brunswick, Fredericton, New Brunswick, Canada; 2 Biology Department, Mount Allison University, Sackville, New Brunswick, Canada; 3 Department of Biology, Carleton University, Ottawa, Ontario, Canada; Institut Pluridisciplinaire Hubert Curien, France

## Abstract

Dispersal, the movement of an individual away from its natal or breeding ground, has been studied extensively in birds and mammals to understand the costs and benefits of movement behavior. Whether or not invertebrates disperse in response to such attributes as habitat quality or density of conspecifics remains uncertain, due in part to the difficulties in marking and recapturing invertebrates. In the upper Bay of Fundy, Canada, the intertidal amphipod *Corophium volutator* swims at night around the new or full moon. Furthermore, this species is regionally widespread across a large spatial scale with site-to-site variation in population structure. Such variation provides a backdrop against which biological determinants of dispersal can be investigated. We conducted a large-scale study at nine mudflats, and used swimmer density, sampled using stationary plankton nets, as a proxy for dispersing individuals. We also sampled mud residents using sediment cores over 3 sampling rounds (20–28 June, 10–17 July, 2–11 August 2010). Density of swimmers was most variable at the largest spatial scales, indicating important population-level variation. The smallest juveniles and large juveniles or small adults (particularly females) were consistently overrepresented as swimmers. Small juveniles swam at most times and locations, whereas swimming of young females decreased with increasing mud presence of young males, and swimming of large juveniles decreased with increasing mud presence of adults. Swimming in most stages increased with density of mud residents; however, proportionally less swimming occurred as total mud resident density increased. We suggest small juveniles move in search of *C. volutator* aggregations which possibly act as a proxy for better habitat. We also suggest large juveniles and small adults move if potential mates are limiting. Future studies can use sampling designs over large spatial scales with varying population structure to help understand the behavioral ecology of movement, and dispersal in invertebrate taxa.

## Introduction

Movement patterns can have profound consequences on the structure and dynamics of populations and communities, and these consequences often depend on which particular stages in the life cycle of an organism are more mobile than others [1]. Studies, mostly on birds and mammals, identified two broad categories of movement: natal dispersal, the movement from the natal area to where breeding first occurs, and breeding dispersal, the movement between two successive breeding areas [1], [2]. Ultimately, dispersal of juveniles appears to be tuned to minimize inbreeding and kin competition and is proximately driven by intraspecific density [3], [4], [5], [6]. Ultimately, dispersal of adults appears to be tuned to maximize mating opportunities with the sex less invested in reproduction dispersing predominantly [3], [7]. Proximate factors influencing adult dispersal include availability of resources, density of the opposing sex, and interactions among these factors [8], [9]. Much progress has been made determining causes of dispersal in vertebrates and marine invertebrate larval stages (adults of marine invertebrate species are typically sessile, resulting in obligate larval dispersal [10], [11]), but similar information on dispersal for invertebrate taxa that are mobile throughout their life history is scarce. While it is known that certain stages of various invertebrate species move more than others (juveniles in marine amphipods [12], [13], [14], adults and juveniles in freshwater stream invertebrates [15], juveniles and sex-biased adults in land-based arthropods [16], [17], [18]), hypotheses regarding the ultimate factors causing these biases have garnered less attention. Examples, however, do include Beirinckx et al. [19], who observed female-biased dispersal in damselflies, and attributed this to maturation rate and foraging behavior in females. Caudill [20] reported female-biased dispersal in mayflies, attributed to the study species’ mating system where males emerge only to swarm for a single day around their natal pond. Albrecsten and Nachman [21] also reported female-biased dispersal in tephritid fly, attributed to pre-emptive competition for oviposition sites and a “sit-and-wait” strategy by receptive males. Baker [22] reported that the isopod *Hemilepistus reaumuri* disperses depending on its body size, with smaller males establishing new burrows more quickly in anticipation of not being able to compete with other males for female initiated burrows.

Knowledge of intraspecific interactions driving dispersal comes largely from long-term tracking studies, focusing on a single or a few populations [7]. These studies rely on temporal fluctuations in population structure and links to variation in dispersal. However, this approach is difficult to apply to invertebrates because individuals can be very small and numerous, rendering mark-recapture methods impractical in many cases (especially in marine environments). The inability to monitor individuals over time breaks the link between biological attributes of a population and dispersal (or proxies of dispersal) of individuals. Note that indirect measures of dispersal serve as suitable proxies where direct measures are not feasible. For example, continuity or isolation of populations is routinely approximated through genetic studies [23]. Measuring the moving portion of the population against a background of known population structure can also be used to identify more mobile life history stages [15].

One way to detect patterns of invertebrate dispersal is to characterize biological attributes of different populations over a large spatial extent and test if these attributes relate to the dispersal of certain life history stages. Typically, studying a given species in a large spatial context should yield variation in population dynamics, including differences in total densities and demographic structure. These differences among populations can in turn be tested for associations with the dispersal of individuals within populations. Such associations, if they exist, provide insight on possible explanations for dispersal, which can be used as working hypotheses in future studies that explicitly test these mechanisms. We used this approach to inform hypotheses about the ultimate causes of dispersal in a model marine invertebrate.

### Model Species

The burrow-dwelling amphipod *Corophium volutator* is a common species of intertidal mudflat ecosystems on the North American and European coasts of the North Atlantic, reaching densities of more than 60,000 individuals m^–2^ [24], [25]. North American populations typically produce two apparent generations each summer, one in late May and one in July that overwinters [24], [26]; both generations overlap throughout the summer [27]. *C. volutator* populations are typically female biased (2∶1 or 3∶1), and scarcity of males might limit reproductive output [28], [29]. Mated females brood eggs that hatch into juveniles that are released into the surrounding mud [30]. *C. volutator* is also a prominent swimmer; individuals of all sizes leave burrows during periods of immersion and drift in the water column [27], [31]. Details of swimming have mostly been studied in Europe [31], [32], [33], [34] and to our knowledge have only been investigated at one mudflat in North America [27], [35], [36]. The abundance of swimming *C. volutator* usually displays seasonal, lunar and diel rhythmicity with peaks occurring at night during periods of immersion around the new or full moon, between June and August [31], [33], [34], [35]. Several studies reported the over representation of small juveniles among swimmers [31], [34]. A detailed study examining differences between mud residents and swimmers was conducted by Drolet and Barbeau [27] at one site, who reported important temporal variation in swimmers that occasionally resulted in increased proportions of small juveniles and males swimming, while ovigerous females tended to swim less.

Swimming by *C. volutator* can serve as a proxy for dispersal. *C. volutator* actively controls its entry and time spent in the water column by swimming, or beating its pleopods [37], [38]. Swimming is also combined with passive movement driven by water currents, with individuals entering the water column during periods of peak velocity in tidal currents [32], [39]. Individuals swimming as high as 4 m into the water column may be transported large distances, up to 14.4 km in a single swimming event [39]. As a consequence, the genetic structure of *C. volutator* populations shows high connectivity among mudflats [40]. Given the high degree of tidal entrainment faced by individuals entering the water column, we worked under the assumption that the characteristics of individuals found in the water column at a particular site are representative of individuals that will travel large distances. Dispersal herein is therefore defined operationally by swimming density or propensity to swim (i.e., standardized swimming activity) of amphipods, and concerns movement away from respective populations.

*C. volutator* is an ideal model invertebrate to study biological drivers of dispersal. Although population dynamics vary little within a mudflat [27], they vary substantially among mudflats [24], providing the backdrop for investigating attributes of populations that associate with dispersal. As well, many *Corophiid* amphipods (possibly including *C. volutator*) are successful invasive species [41], highlighting the importance of dispersal to the biology of such amphipods. Here, we expand on previous population studies by considering the demographics of the dispersing subset in greater detail. By studying multiple mudflats simultaneously throughout the upper Bay of Fundy (spanning an approximate area of 10,000 km^2^), we observed dispersal under a broad range of population states (density, size structure and sex structure). Our first objective was to confirm that most variation in swimming occurs at the level of site (a.k.a. mudflat) to ensure we had appropriate variability to assess differences in swimmers over different population states. Our second objective was to identify the main life history stages of dispersal by determining which sizes or sexes of individuals were swimming relative to non-swimming individuals. Our third main objective was to use the among-site variation in swimming individuals to identify potential population factors that may drive dispersal. For this objective, we assessed if dispersal of different life history stages varied in relation to overall density (or biomass) of mud residents. We also assessed whether the swimming propensity of certain stages associated with certain characteristics of the mud resident population. More specifically, we examined (i) whether standardized swimming activity (density of a swimming stage standardized by its density in the mud) of juveniles correlated with the density of resident (mud) adults; (ii) whether standardized swimming activity of males correlated with resident density of females of different reproductive stages, and (iii) whether standardized swimming activity of non-ovigerous females correlated with resident density of males. Observing resident and swimming individuals at several mudflats spanning the upper Bay of Fundy provided a robust ground to better describe the dispersal behavior of *C. volutator,* which in turn allowed us to provide insights on possible mechanisms driving this behavior. By informing hypotheses regarding the dispersal of *C. volutator*, we hope to illustrate possible avenues for mechanistic models that would further our understanding of dispersal in invertebrate taxa.

## Methods

### Sampling of Swimmers

No specific permits were required for the described field studies, as we sampled non-protected marine invertebrates (amphipods, annelids, gastropods, bivalves, nematodes) in non-privately-owned or non-protected locations (intertidal mudflats located below the low high tide line, according to Canadian guidelines).

**Figure 1:**
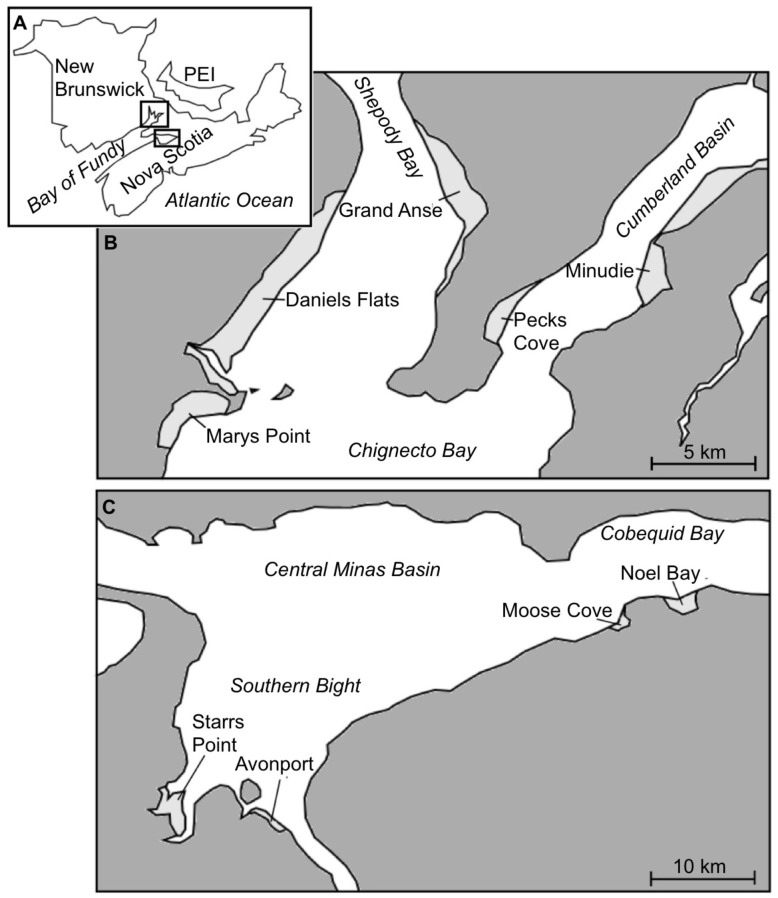
Site locations. **A** Maritime provinces of Canada, showing the location of the Bay of Fundy; the rectangles represent Chignecto Bay and the Minas Basin. **B** and **C** detail the two main branches of the upper Bay of Fundy. Major mudflats are shown in light grey and the sites where sampling was conducted are identified.

**Figure 2:**
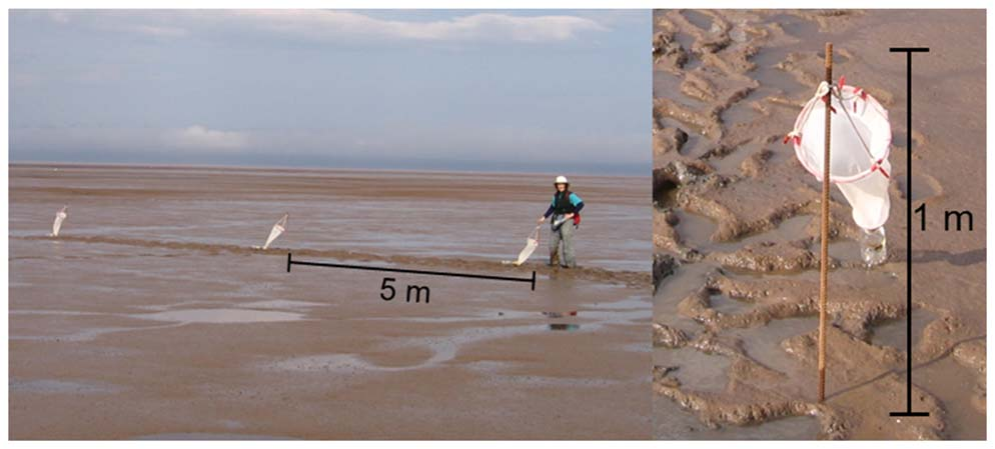
Plankton nets deployed at Daniels Flats mudflat, New Brunswick, summer 2010. The triplicate nets were aligned parallel to the low water line. The tidal height at this particular location is ∼5 m above the mudflat surface. Photographs by KR Richard.

To evaluate spatial and temporal patterns in density and population structure of swimming *C. volutator*, we sampled the water column with stationary plankton nets at 9 mudflats during summer 2010. Site locations spanned throughout the upper Bay of Fundy in the Chignecto Bay (Marys Point, Daniels Flats, Grand Anse, Pecks Cove, Minudie) and Minas Basin regions (Noel Bay, Moose Cove, Avonport, Starrs Point; Figure 1), where tidal amplitude is amongst the highest in the world (up to 16 m). The sites selected represent many of the major mudflats in the upper Bay of Fundy, ranging in size from 1.4 to 17 km^2^ (6.0±4.6 km^2^, mean±SD, n = 9 mudflats).Three plankton nets were deployed at each site in a line parallel to the low water line with 5 m between adjacent nets (Figure 2). The nets were secured at the top of metal posts, 1 m above the substratum, and were free to rotate with the water current direction. We placed the nets at a fixed elevation of 7.5 m above 0 chart datum at 8 mudflats (which is a distance of 50–300 m from shore); elevation was measured with surveying equipment. The nets at Starrs Point were placed at an elevation of 11.7 m (and 20 m from shore); this mudflat has a very shallow incline, and hardly dips below 10.5 m in elevation 500 m onto the mudflat. These nets were, however, immersed each sampling round; at 7.5 m elevation, the nets were submerged for most of the high tide cycle (∼4 h). A plankton net consisted of a metal ring (20 cm diameter) at the mouth of the net, and fabric (180 µm mesh size, which captures all sizes of *C. volutator* [42]) extending 60 cm to the narrow end of the net, with a plastic funnel (with an 8 cm diameter opening) glued at the narrow end of the net (Figure 2) [35].

Sampling was conducted three times during summer 2010 (25–28 June, 10–13 July and 8–11 August) for three consecutive nights bounding new or full moons. Plastic bottles (500 ml, each with a 2 cm x 4 cm window covered with 180 µm mesh to prevent pressure build-up) were attached to the funnel of the nets each evening before immersion of the sites, and samples were collected in the morning following emersion. The samples were rinsed in a 250 µm sieve [42] stored in 95% ethanol, and later processed using a dissecting microscope. The number of individuals, and size (body length from tip of rostrum to end of telson) and sex of adults (>4 mm in body length; sexing detailed by Schneider et al. [28], and Barbeau and Grecian [43]) were recorded. Intersex individuals (consisting of 2.0±0.5% [mean±SE, n = 241 samples] of swimming adults) were pooled with males, since they are functional males [44]. A Folsom plankton splitter was used to subsample (1/2 to 1/512 subsamples) when an original sample exceeded 200 individuals to have 100–200 individuals to process. The number of individuals per sample was converted to a density (ind. M^–3^) by dividing by the volume of water filtered through each net. The volumes of water were estimated using dissolution of plaster hemispheres [45] attached at the mouth of each net; the procedure was calibrated using a saltwater flume [27].

### Sampling of Mud Residents

We used mud core samples collected at each of our sites to sample mud residents. At each site, we sampled along a transect extending the eulittoral zone of the mudflat (range of transect lengths: 700 to 1800 m). Core samples were collected at 12 predetermined stratified random locations along each transect, using a 7 cm diameter corer, which was pushed into the mud to the depth of the compact hypoxic layer (3–6 cm deep). The mud was sampled: 20–24 June, 14–17 July and 2–7 August; only two mudflats (one randomly chosen per region [Chignecto Bay and Minas Basin]) could be sampled per day (we had two sampling teams working concurrently). Samples were rinsed in a 250-µm sieve and stored in 95% ethanol. *C. volutator* were processed from the samples as described above, though mud samples were not subsampled.

### Data Analysis

Our first objective was to verify that variation in swimming *C. volutator* occurs at large spatial scales (i.e. level of mudflats). We used ANOVAs with the random factors of Site (9 mudflats), Round (3 sampling rounds: June, July and August), and Night (3 sampling dates per round) nested in Round. The structure of the ANOVA is presented in Table 1, and appropriate denominators for F-ratios and Quasi F-ratios were determined [46], [47]. Variance component analysis was done to estimate the proportion of variation explained by the different sources of variation [47]. This analysis was used to examine several dependent variables: total density of swimmers (ind. M^–3^ of water), densities of different size classes (<1.5, 1.5–2.5, 2.5–4, 4–6 and >6 body length) and densities of adult males, non-ovigerous females, and ovigerous females. Density variables were transformed using log10(datum+1) to correct for heterogeneity of variance.

**Table 1:** Structure of the random model ANOVA used in the analysis of densities of swimming *Corophium volutator* in the upper Bay of Fundy.

Source of variation	F-ratio or Quasi F-ratio	Variance component
Round R*_i_*	(MS*_R_*+MS*_SN(R)_*)/*(*MS*_N(R)_*+MS*_RS_*)	(MS*_R_*+MS*_SN(R)_*−MS*_RS_*−MS*_N(R)_*)/*acn*
Site S*_j_*	MS*_S_*/MS*_RS_*	(MS*_S_*−MS*_RS_*)/*bcn*
Round x Site R*_i_*S*_j_*	MS*_RS_*/MS*_SN(R)_*	(MS*_RS_*−MS*_SN(R)_*)/*cn*
Night(Round) N*_k_*(R*_i_*)	MS*_N(R)_*/MS*_SN(R)_*	(MS*_N(R)_*−MS*_SN(R)_*)/*an*
Site x Night(Round) S*_j_*N*_k_*(R*_i_*)	MS*_SN(R)_/*MS*_e_*	(MS*_SN(R)_*−MS*_e_*)/*n*
Error e*_l(ijk)_*		MS_e_

For the variance components, *a* is number of sites, *b* is number of sampling rounds, *c* is number of nights sampled within a sampling round, and *n* is the harmonic mean of number of replicate samples in a night. MS = mean square.

To identify dispersal stages in *C. volutator*, we compared the size distribution of swimmers (0.5 mm increments, from 1 to 11 mm in body length) to that of the mud resident using bootstrap randomization procedures. We assumed that the expected size distribution of swimmers is a random subsample of the mud residents. For each site and round, we generated 95% confidence intervals around the size distribution of mud residents (i.e., the expected distribution based on 12 pooled cores). We randomly selected individuals out of the mud resident size distribution 1000 times and removed the 25 smallest and largest bootstrap values for each size class to get the lower and upper confidence limits. For each iteration, the number of randomly-selected individuals was the actual number of individuals measured in the plankton net samples. The observed proportions for swimmers (means from 7–9 nets) were then compared to the expected proportions, and size classes having observed proportions lying outside of the confidence interval limits of the expected distributions were declared significantly different. This analysis was done only for site-round combinations with at least 100 swimming individuals and 100 mud residents. To compare the stage structure of swimming adults (i.e., observed values) to mud resident adults (i.e., expected values), we used G-tests [48] with five amphipod stages: small and large males (4–6 mm and >6 mm), small and large non-ovigerous females (4–6 and >6 mm), and ovigerous females (which ranged between 4.7 and 10.8 mm). We computed the percentages of adults in each stage for Site*Round combinations with >100 total mud residents (pooled over 12 mud cores) and >100 individual swimmers pooled over 3 nights (n = 9 nets). If a stage had 0 individuals, it was pooled with another appropriate stage; specifically, large males were pooled with small males, and large females were pooled with ovigerous females (given that small males mature into larger males and that ovigerous females were frequently the largest individuals).

To gain insight on potential biological drivers of dispersal in *C. volutator*, we tested the effect of mud resident density (or biomass) on densities of swimming individuals using Model II regressions on log-transformed data. Specifically, reduced major axis regression analysis was used to account for variation in the estimate of our independent variable (Site*Round-averaged densities [ind. M^–2^] or biomasses [mg m^–2^] of total mud residents; each estimate was calculated from 12 core samples) [48], [49], [50]. The dependent variables were Site*Round-averaged densities of swimmers (total density and densities according to size class [<1.5, 1.5–2.5, 2.5–4, 4–6, >6 mm body length] or adult stage [males, non-ovigerous females and ovigerous females]). We first tested the slope for each regression against the value of 0 using a standard Student’s t-test (to determine if there was a significant relationship). If we had a significant relationship (i.e., slope different from 0), then we used Clark’s T-test to determine if the slope was significantly different from 1. Determining if a significant slope is greater or smaller than 1 is relevant because this indicates the type of relationship between the two variables (Figure S1). If the slope (on a log-log scale) is smaller than 1, then we have an ascending but decelerating relationship, suggesting a decreasing proportion of swimming individuals as mud density increases. If the slope equals 1, then a constant proportion of individuals swim across mud densities. Finally, if the slope is greater than 1, then an increasing proportion of individuals swim with increasing density of residents (ascending accelerating relationship). This analysis was done with two measures of *C. volutator* abundance in the mud as the independent variable, density (number of individuals m^–2^, which reflects the amount of mudflat surface inhabited by *C. volutator*) and biomass (mg m^–2^, which reflects the total mass of *C. volutator* within a site). Biomass per sample was calculated from the length-weight relationship in Boates and Smith [51].

To determine if the propensity to swim of certain stages of *C. volutator* is related to the structure of mud resident population, we used Pearson correlation analysis between standardized swimming activity for a stage of interest and density of a mud resident stage of interest. Because each correlation tests a different null hypothesis, our Type 1 error rate would not be inflated (which is a problem when repeatedly testing the same hypothesis). Standardized swimming activity was calculated by dividing the density of a given swimming stage by its respective density in the mud, thus providing a measurement of swimming activity relative to availability in the mud. All variables were calculated using the averages (n = 9 nets for swimmers or n = 12 mud cores for residents) for each Site*Round combination. Swimming stages investigated included small, medium and large juveniles (<1.5, 1.5–2.5 and 2.5–4 mm body length, respectively), total males, small and large males (4–6 and >6 mm, respectively), total females, and small and large non-ovigerous females (4–6 and >6 mm, respectively). The mud resident variables were total adult density for the questions about juvenile swimming, densities of different reproductive stages of females (i.e., total females, total non-ovigerous females, small and large non-ovigerous females, and ovigerous females) for questions about male swimming, and densities of males (i.e., total males, and small and large males) for questions about female swimming.

## Results

### Does the Density of Swimmers Vary Spatially?

**Figure 3:**
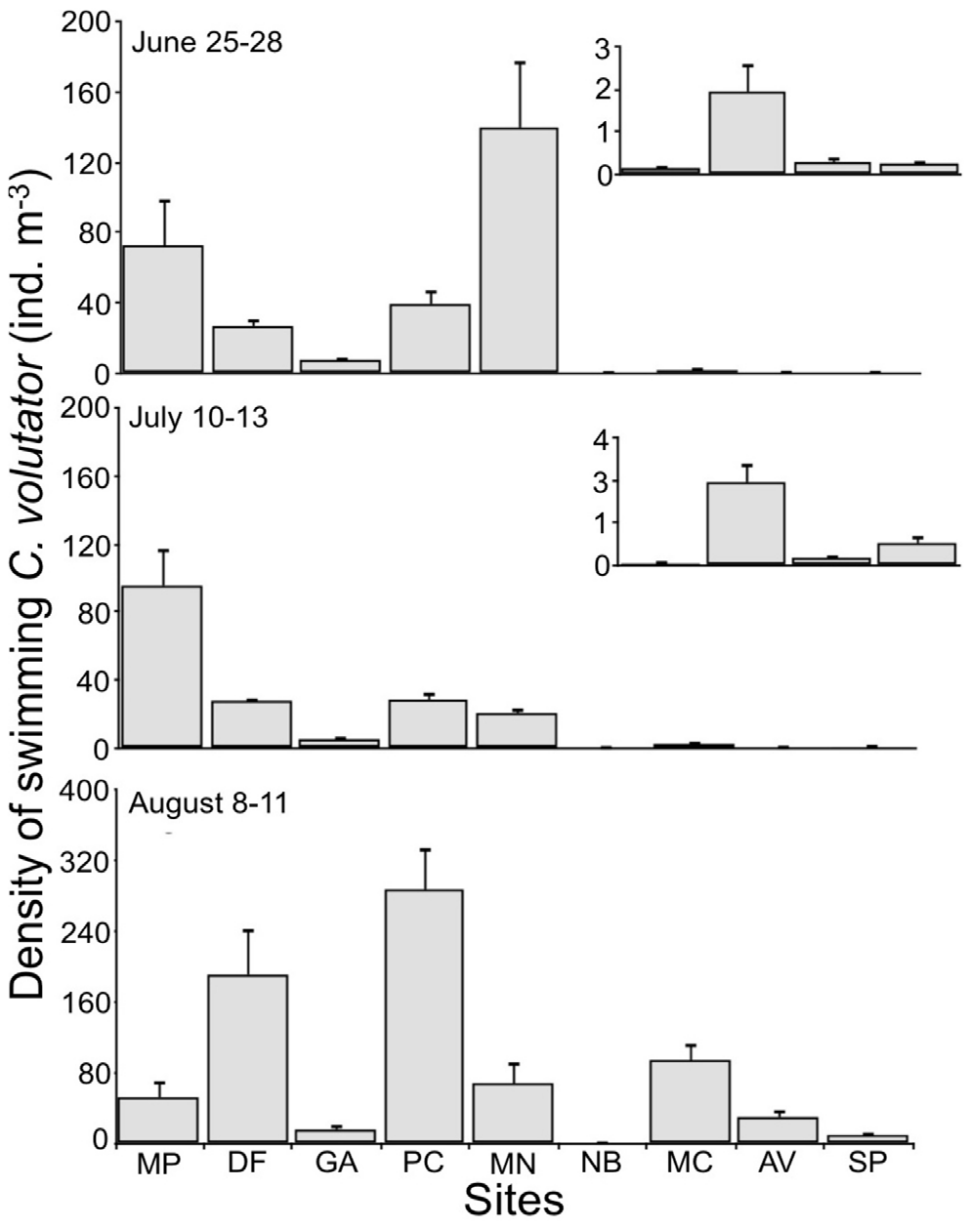
Mean (+ SE) density of swimming *Corophium volutator* at each site, summer 2010. Data are for sites in the upper Bay of Fundy during June, July and August sampling rounds: MP = Marys Point, DF = Daniels Flats, GA = Grand Anse, PC = Pecks Cove, MN = Minudie, NB = Noel Bay, MC = Moose Cove, AV = Avonport, SP = Starrs Point. n = 7–9 nets (2–3 replicate nets over each of 3 replicate nights. The inserts for June and July are magnified charts for the Minas Basin sites. The density for Noel Bay in August was 0.27±0.06 ind. M^–3^.

Swimming occurred throughout the upper Bay of Fundy, although considerably less occurred in the Minas Basin than in Chignecto Bay (Figure 3). Spatial variation in the density of swimmers was most important at the large scale, with the factor site accounting for most of the variation in total density (Table 2). Similar patterns were observed when considering size and sex classes (Table S1).

**Table 2:** ANOVA results for total density (ind. M^–3^, log_10_[datum+1]-transformed) of swimming *Corophium volutator* in the upper Bay of Fundy, summer 2010.

Source of variation	df	MS	F	p	Variance component	% of Variation
Round	2	7.282	3.6	0.048	0.068	9.7
Site	8	11.666	10.5	<0.001	0.396	56.3
Round x Site	16	1.112	4.5	<0.001	0.097	13.8
Night(Round)	6	0.969	3.9	0.003	0.027	3.8
Night(Round) x Site	48	0.248	5.3	<0.001	0.068	9.7
Error	160	0.047			0.047	6.7

All sources of variation are random.

### Does the Stage Structure of Swimmers Reflect that of Mud Residents?

**Figure 4:**
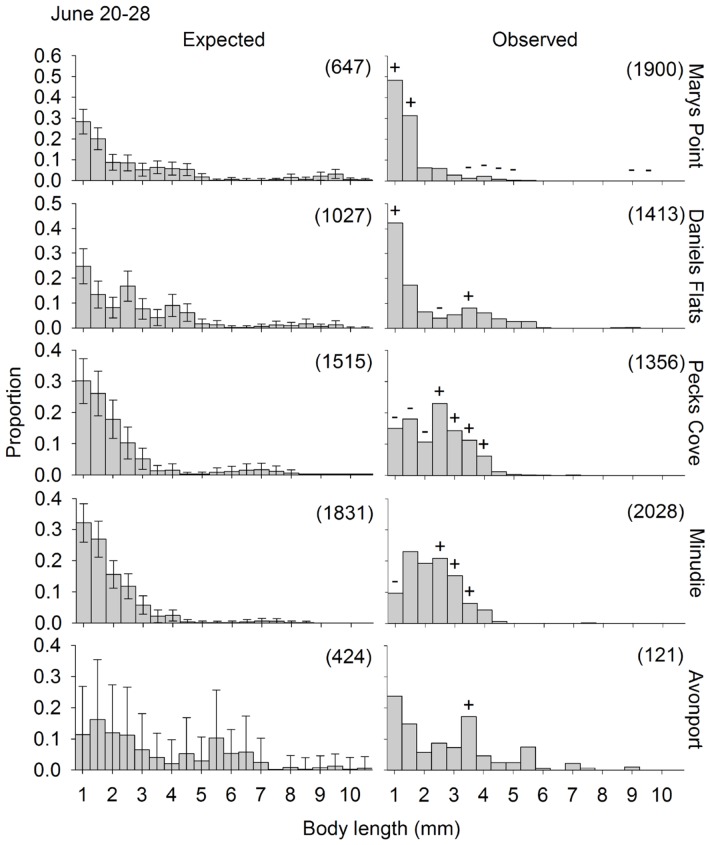
Observed and expected size distributions of *Corophium volutator* individuals swimming in the water column. Distributions are for sites in the upper Bay of Fundy during June 2010. The expected proportions correspond to the observed proportions residing in the mud (pooling over 12 cores). Error bars are 95% confidence intervals obtained by subsampling (bootstrapping) the mud resident population. The observed proportions for swimmers are the mean, calculated from the 9 nets (3 replicate nets for each of 3 replicate nights). Symbols above bars in an observed distribution indicate 0.5-mm size classes which had a greater (+) or smaller (−) proportion swimming than expected (based on the confidence intervals of the expected distribution). The numbers in parentheses represent the number of individuals measured to obtain the size distributions.

**Figure 5:**
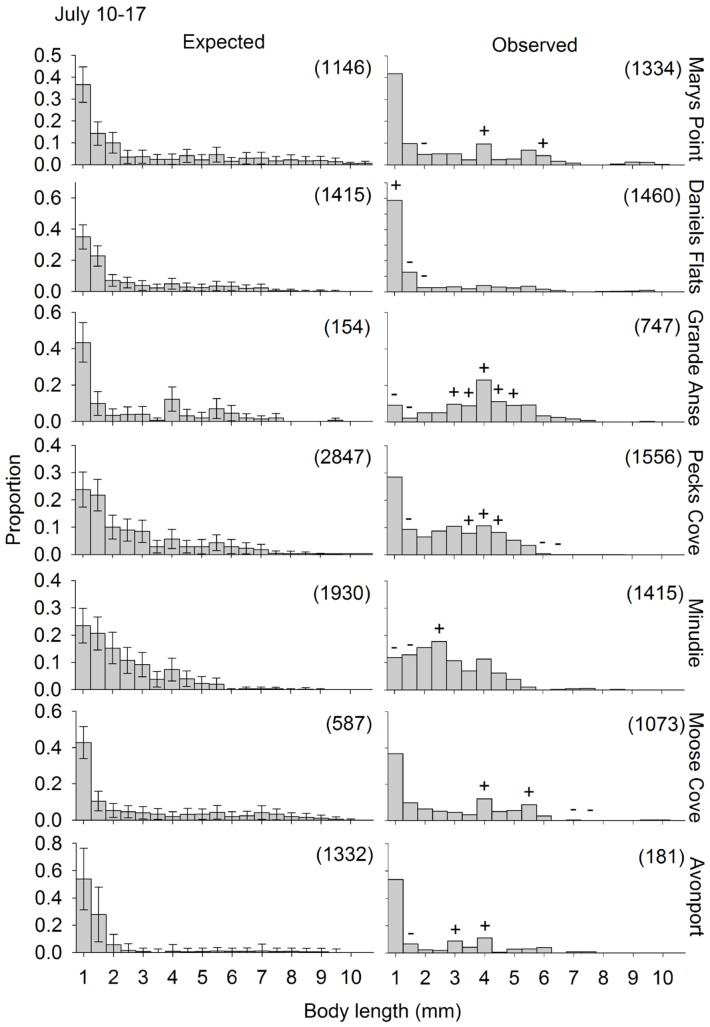
Observed and expected size distributions of *Corophium volutator* individuals swimming in the water column. Distributions are for sites in the upper Bay of Fundy during July 2010. The expected proportions correspond to the observed proportions residing in the mud (pooling over 12 cores). Error bars are 95% confidence intervals obtained by subsampling (bootstrapping) the mud resident population. The observed proportions for swimmers are the mean, calculated from the 9 nets (3 replicate nets for each of 3 replicate nights). Symbols above bars in an observed distribution indicate 0.5-mm size classes which had a greater (+) or smaller (−) proportion swimming than expected (based on the confidence intervals of the expected distribution). The numbers in parentheses represent the number of individuals measured to obtain the size distributions.

**Figure 6:**
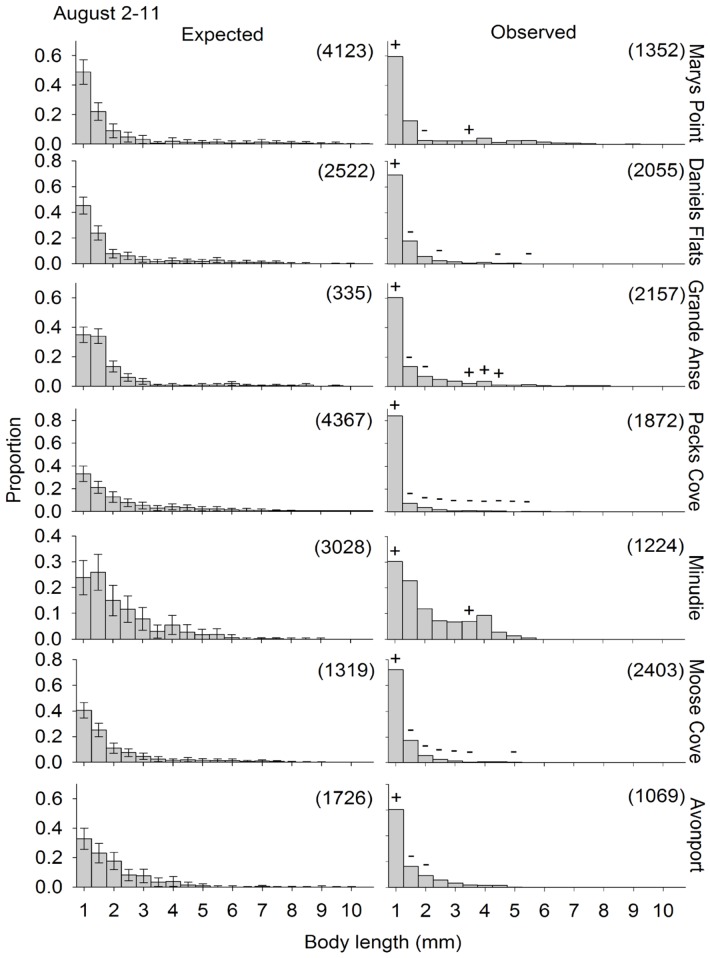
Observed and expected size distributions of *Corophium volutator* individuals swimming in the water column. Distributions are for sites in the upper Bay of Fundy during August 2010. The expected proportions correspond to the observed proportions residing in the mud (pooling over 12 cores). Error bars are 95% confidence intervals obtained by subsampling (bootstrapping) the mud resident population. The observed proportions for swimmers are the mean, calculated from the 7–9 nets (2–3 replicate nets for each of 3 replicate nights). Symbols above bars in an observed distribution indicate 0.5-mm size classes which had a greater (+) or smaller (−) proportion swimming than expected (based on the confidence intervals of the expected distribution). The numbers in parentheses represent the number of individuals measured to obtain the size distributions.

**Figure 7:**
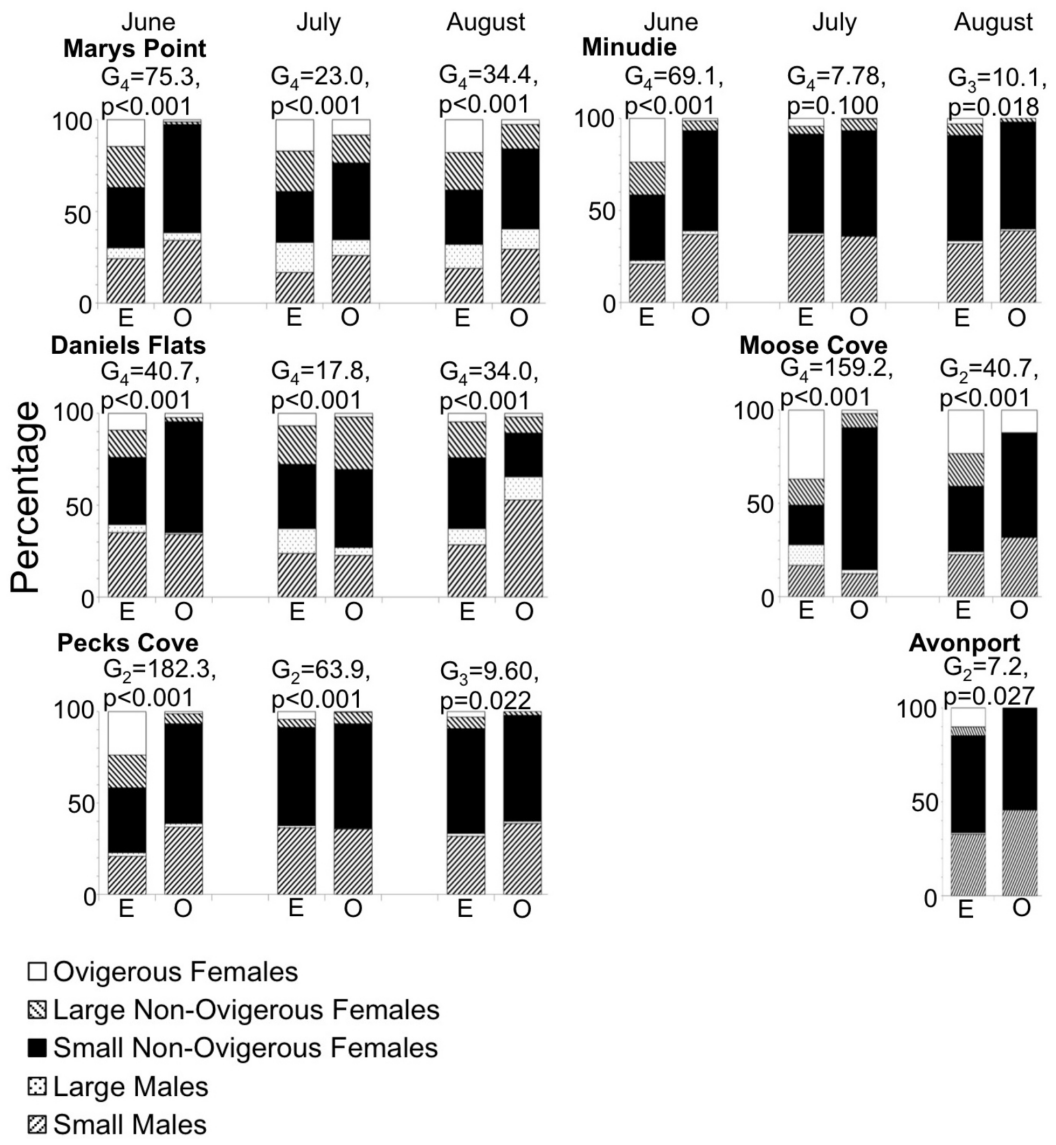
Percentages of different stages of *Corophium volutator* adults in the mud (expected, E) and swimming (observed, O). Data are for sites in the upper Bay of Fundy, summer 2010. The G-test statistic (subscript = degrees of freedom) and associated p-value compare expected (mud resident) and observed (swimming) distributions. For expected values, data were pooled over 12 cores; for observed values, the 2–3 nets per night were pooled, and the 3 replicate nights averaged. The df in the G-test is <4, when one or more adult stages with 0 individuals needed to be pooled with another stage. Site-round combinations with less than 100 *C. volutator* adults in the mud were not analysed. Small males and small non-ovigerous females are 4–6 mm body length, and large males and large non-ovigerous females are >6 mm. Ovigerous females ranged from 4.7 to 10.8 mm.

The observed size distribution of swimmers was different from the expected size distribution (if swimmers are a random subsample of the residents) for all site and round combinations for which the randomization test could be performed (Figures 4, 5, 6). One of two distinct patterns was apparent for all site and round combinations. First, the smallest size class (1–1.5 mm) was often overrepresented in the plankton samples, at a few sites in June and July (Figures 4 and 5, respectively), and at all the sites in August (Figure 6). Second, one or several of the intermediate size classes (large juveniles and small adults) were overrepresented in the plankton samples, which generally occurred when the small juveniles were not overrepresented (11 out of 15 times). The other times, both small juveniles and large juveniles were overrepresented. The stage structure of adult swimmers differed significantly from that expected based on adult residents in all Site*Round combinations tested, except Minudie in July (Figure 7). In nearly all cases that could be tested and were significant, large adults (males and non-ovigerous females >6 mm) and ovigerous females swam less than expected. Where significant, small adult males (4–6 mm) often swam more than expected, and small non-ovigerous females almost always swam more than expected (with 5 exceptions for males, and 1 exception for females).

### How does Swimming (or Potential for Dispersal) Relate to Density or Biomass of Mud Residents?

**Figure 8:**
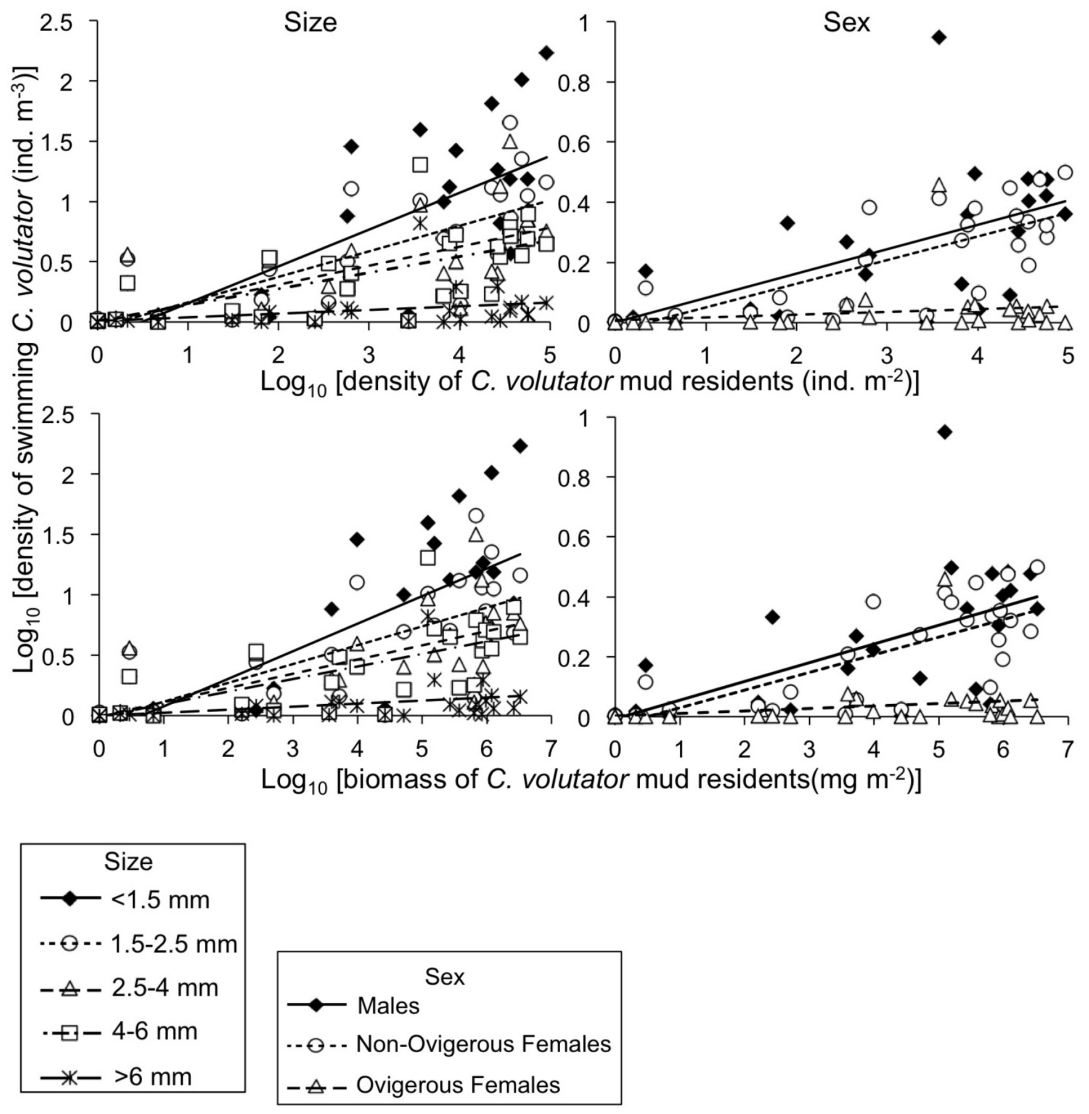
Densities of swimming *Corophium volutator* versus total mud density or biomass of *C. volutator*. Data are for various *C. volutator* size and sex classes, and are site-round combinations in the upper Bay of Fundy, summer 2010. For swimmer values, mean of 7–9 nets (2–3 replicate nets for each of the 3 replicate nights); for mud resident values, mean of 12 replicate cores. See Table 3 for results of statistical analyses.

Swimming density of most stages of *C. volutator* was positively related to the overall abundance of residents, measured both as density (number of individuals m^–2^) and as biomass (mg m^–2^), as indicated by a positive slope significantly different from 0 (Table 3, Figure 8). The relationship was not significant for large adults (>6 mm) and ovigerous females. Significant slopes were shallowest for adults and steepest for small juveniles (<1.5 mm). However, all significant slopes were significantly less than 1, indicating a swimming response of most stages that is density dependent, with a decelerating rate of increase (Figure S1).

**Table 3:** Model II regression results for swimming *Corophium volutator* variables tested against total density or biomass of *C. volutator* residents in the mud in the upper Bay of Fundy, summer 2010.

Dependent variable (swimming density, ind. m^-3^)	Slope	±SE of slope	r	df	Student’s t-test, H_o_: Slope = 0	p	df	Clark’s T-test, H_o_: Slope = 1	p
For total density in the mud (ind. M^–2^) as the independent variable									
Total	0.37	0.06	0.78	26	6.3	<0.001	21.2	3.4	0.002
<1.5 mm	0.30	0.06	0.73	26	5.3	<0.001	21.8	3.8	0.001
1.5–2.5 mm	0.22	0.04	0.72	26	5.2	<0.001	21.9	4.7	<0.001
2.5–4 mm	0.18	0.04	0.66	26	4.4	<0.001	22.5	5.0	<0.001
4–6 mm	0.15	0.03	0.66	26	4.4	<0.001	22.6	5.4	<0.001
>6 mm	0.06	0.02	0.31	26	1.6	0.120			
Male (>4 mm)	0.10	0.02	0.59	26	3.6	0.001	23.3	6.2	<0.001
Non-ovigerous female (>4 mm)	0.08	0.01	0.77	26	6.0	<0.001	21.3	8.6	<0.001
Ovigerous female	0.03	0.01	0.17	26	0.9	0.388			
For total biomass in the mud (mg/m^2^) as the independent variable									
Total	0.28	0.04	0.76	26	5.9	<0.001	21.4	4.3	<0.001
<1.5 mm	0.23	0.05	0.71	26	5.0	<0.001	22.0	4.6	<0.001
1.5–2.5 mm	0.17	0.03	0.69	26	4.8	<0.001	22.2	5.4	<0.001
2.5–4 mm	0.13	0.03	0.65	26	4.2	<0.001	22.7	5.7	<0.001
4–6 mm	0.12	0.02	0.67	26	4.5	<0.001	22.5	6.3	<0.001
>6 mm	0.04	0.01	0.33	26	1.7	0.093			
Male (>4 mm)	0.08	0.02	0.59	26	3.7	0.001	23.3	7.0	<0.001
Non-ovigerous female (>4 mm)	0.06	0.01	0.75	26	5.7	<0.001	21.5	9.4	<0.001
Ovigerous female	0.02	0.01	0.20	26	1.0	0.309			

Both swimmer and mud resident variables were log_10_-transformed prior to analysis. Student’s t-test evaluates if the slope is significantly different from 0, while Clark’s T-test evaluates if the slope is significantly different from 1 (MacArdle 1988). r = correlation coefficient.

### Is the Propensity to Swim of Certain Stages Related to Other Stages of Mud Residents?

**Figure 9:**
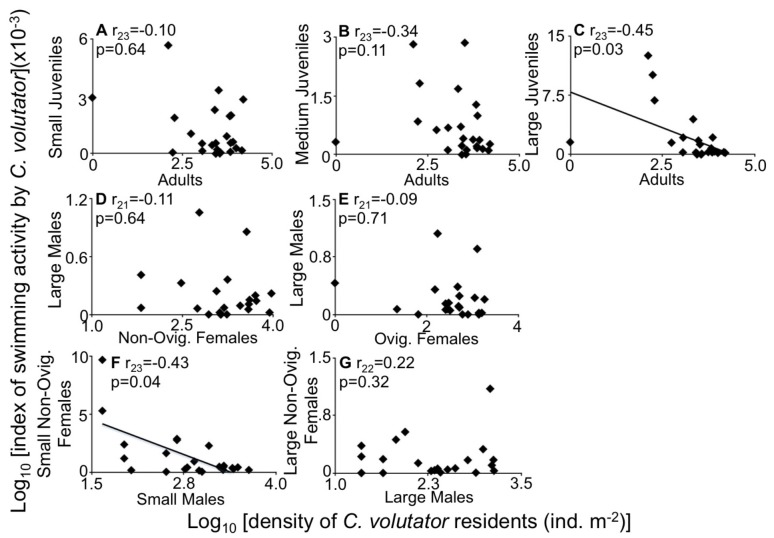
Standardized swimming activity of *Corophium volutator* plotted against a density variable for the mud residents. Data are site-round combinations in the upper Bay of Fundy, summer 2010. Standardized swimming activity was calculated as density of swimmers [m^–3^] divided by the respective density of mud residents [m^–2^]. For swimmer values, mean of n = 7–9 nets; for mud resident values, mean of 12 replicate cores. Small juveniles <1.5 mm body length, medium juveniles = 1.5–2.5 mm, large juveniles = 2.5–4 mm, small males and small non-ovigerous females = 4–6 mm, large males and large non-ovigerous females >6 mm. Ovigerous females ranged between 4.7 and 10.8 mm. The correlation coefficient (r, subscript = degrees of freedom) and p-value for Pearson’s correlation test on the untransformed data are presented; for significant correlations, the line of best fit is plotted.

Standardized swimming activity of certain stages was significantly associated with characteristics of the mud resident population for two stages (Figure 9, Table S2): large juveniles and small non-ovigerous females (2.5–4 and 4–6 mm respectively, Figure 9C, F). Standardized swimming activity of large juveniles was negatively correlated to density of resident adults (Figure 9C), indicating that these juveniles are less likely to swim at higher adult densities in the mud. However, standardized swimming activity of smaller juveniles (<1.5, 1.5–2.5 mm) was not associated with density of resident adults (Figure 9A, B). Standardized swimming activity of males (whether it be all males, small males or large males) was not strongly associated with any resident female features (densities for all females, non-ovigerous females or ovigerous females; p>0.08; Figure 9D, E; Table S2). Standardized swimming activity of small non-ovigerous females (4–6 mm) was negatively correlated with small resident males (4–6 mm; Figure 9F). No other significant associations were found between swimming propensity of females and any other resident male features (p>0.09; Table S2).

## Discussion

The mobility, abundance, and broad diversity of life histories of invertebrates can provide invaluable systems to test and refine hypotheses about dispersal, yet methodologies to accurately study such small animals remain scarce. Here, we used large spatial scale variation in population structure of both mud residents and swimmers to provide insight on mechanisms of dispersal in a model invertebrate species.

### Spatial Variation in Density of Swimmers

Studies of swimming behavior in a key mudflat species *Corophium volutator* led to a better understanding of its population distribution and maintenance [27], [36], [39], [52]. However, the adaptive significance of dispersal in this species remained poorly understood. Prior to our study, swimming of *C. volutator* was only investigated at one site in the Bay of Fundy (Pecks Cove); our first objective was to evaluate variability in swimming across mudflats. Our results clearly demonstrated that swimming occurs at all the sites sampled, which correspond to many of the major mudflats of the upper Bay of Fundy (Figure 3). Most of the variation in the density of swimmers occurred among mudflats, notably between the two basins (Chignecto Bay and Minas Basin). Population structure of individuals in the mud can differ greatly among mudflats [24], and so can that of swimmers among mudflats. Distinct population structure of swimmers, even for mudflats adjacent to one another, suggests that movement by individuals largely occurs within the boundaries of mudflats. However, high rates of gene flow suggest that dispersing individuals are reaching adjacent mudflats [40]; individuals found in the water column are most likely representative of those individuals dispersing large distances [39].

### Dispersal Stages

Individuals of all stages can disperse, but marked differences in the degree of swimming occurred among stages. We identified two main stages for dispersal: the smallest juveniles are prominent dispersers, swimming at most times and places, and large juveniles/small adults (particularly females) disperse under certain conditions. Both stages are pre-reproductive (or starting to reproduce), and so correspond to juvenile dispersal [1]. In contrast, large adults, presumably individuals who have already produced offspring, dispersed relatively little, meaning that true adult dispersal likely does not occur in *C. volutator*. This apparent lack or low level of adult dispersal may be a consequence of the life history of *C. volutator,* which usually live through a single breeding season [24].

### Factors Driving Dispersal of Small Juveniles

Juvenile dispersal occurs when individuals move away from their place of birth, but the evolutionary underpinnings of this behavior remain debated [3]. *C. volutator* exhibited juvenile dispersal as recently hatched individuals (Figure 6); Stevens et al. [14] also reported significant net movements of small (<2.2 mm) juvenile corophiid amphipods in a New Zealand estuary. Dispersal by the smallest juveniles in our study did not appear to be driven by intraspecific competition: we did not detect positive density dependence (which would be an ascending accelerating relationship; Figures 8 and S1, Table 3) and there were no associations between the propensity to swim of young juveniles and presence of adults (Figure 9A). One hypothesis suggested by the observed ascending but decelerating rate of dispersal with increasing mud density, is that *C. volutator* seeks high densities of individuals in the mud, particularly early in life (Figure 8, Table 3); gregarious behavior has been previously observed in *C. volutator* [53], [54], though movement from high to low density patches was reported from mesocosm experiments [55], [56], and field experiments [57, 58; see Drolet et al. (54) for possible explanations for differences in results]. If *C. volutator* exhibits gregarious behavior, it is possible that high mud density is a good proxy for habitat suitability, though what constitutes “good habitat” needs to be examined using measured environmental variables beyond population structure (e.g. abundance of diatom food [59], presence/absence of predators [60]). The tendency to aggregate may also benefit the individual by facilitating mate finding earlier in life. On the other hand, the lack of an association between the dispersal of the smallest juveniles and surrounding population structure may indicate that dispersal early in life, if not precipitated by environmental factors, is a “hard-wired” behavior; the possibilities of inbreeding or kin competition avoidance, though difficult to discern, could be investigated [61], [62]. These hypotheses require further testing.

### Factors Driving Dispersal of Larger Individuals

Sexually mature individuals typically disperse to increase probabilities of finding a mate [2], [7]. Dispersal by *C. volutator* seems in tune with ideas about reproductive opportunities. Increased dispersal was observed with the onset of adulthood by large juveniles and small adults, especially females, possibly driven by mate limitation. Similar to small juveniles, swimming of large juveniles and small adults was not positively density dependent (or exhibiting intraspecific competition; Figure 8, Table 3). However, intraspecific interactions appeared to exist between these individuals and other mud resident stages. The propensity to swim of large juveniles was negatively associated with the presence of resident adults (Figure 9C), meaning that large juveniles swam proportionally less as adult presence increased in the mud. Adult presence would be a good indicator of the presence of potential mates, and so large juveniles from areas with low adult presence might be dispersing to secure mating opportunities. Dispersal by small adults might also be a mechanism for mate finding. Small females dispersed more than expected (Figure 7) and the propensity to swim of small females was negatively associated with the presence of mud resident small males (Figure 9F). The tendency for females to disperse substantially could be a reflection of the skewed sex ratio present in *C. volutator* populations, where females typically outnumber males 2∶1 or 3∶1 [24], [26], and in extreme cases 20∶1 [27]. The lower relative abundance of males leads to a smaller proportion of females becoming ovigerous and to reduced brood size, and so females may compete for males [29]. Therefore, presence of males may be an important factor in driving dispersal at the onset of sexual maturity. Our results suggest that females disperse to maximize their chances of finding a mate in a limited pool of males. If this hypothesis is true, males may be maximizing their reproductive output and minimizing their own risks (mortality through dispersal) by simply allowing females to find them. Female-biased juvenile dispersal has been reported in the corophiid amphipod *Paracorophium spp*. [14] and female-biased adult dispersal has been reported in several insects [18], [19], [20].

### Conclusions

We have demonstrated the importance of studies at large spatial scales, focused on population variation, when studying invertebrate dispersal. By expanding sampling of swimming *C. volutator* from one site to several major mudflats in the Bay of Fundy, we identified *C. volutator’s* main dispersal stages and offered possible explanations as to why *C. volutator* disperses. Small juveniles (<1.5 mm) disperse regardless of surrounding population structure; the implications or reasons for aggregating in the mud as young juveniles should be investigated. In addition, we found associations between the propensity to disperse in large juveniles (2.5–4 mm) and small females (4–6 mm) and the density of mud resident adults and males, respectively. Our findings suggest that *C. volutator* on the cusp of reproduction disperse in response to mating requirements, and that females often assume the role of dispersal given sex-ratios are skewed in their favor. The observations we reported required variation in resident population structure as a backdrop to our sampling of dispersing individuals, and future studies can follow up on these observations and test for behavioral mechanisms of invertebrate dispersal. Future studies could also adopt our sampling design and statistical approach to identify dispersal stages in other invertebrate species, which will in turn enhance understanding of dispersal over a wider range of taxa; the necessity of large spatial contexts should not be overlooked.

## Supporting Information

Figure S1 **Linear and exponential (increasing at an accelerating rate and increasing at a decelerating rate) relationships in a) untransformed space and b) in log_10_ transformed space.** The slopes for log-transformed lines are indicated. If the x-axis represents population density, the density dependent relationships can be thought of in a similar way to functional responses in predation theory [63].
(TIF)

Table S1 ANOVA results for densities (ind. m^-3^, log_10_[datum+1]-transformed) of swimming *Corophium volutator* in the upper Bay of Fundy, summer 2010. All sources of variation are random. The degrees of freedom are the same as in Table 2.
(DOCX)

Table S2 Results for correlation analyses between standardized swimming activity and mud resident variables of *Corophium volutator*. Significant results are bolded. Patchiness was calculated as variance/mean for the given variable. Body lengths are in mm.
(DOCX)
