# Supplementary figures and images for: Spatial Variation in Population Structure and Its Relation to Movement and the Potential for Dispersal in a Model Intertidal Invertebrate
Source: PLoS One. 2013 Jul 12;8(7):e69091. doi: 10.1371/journal.pone.0069091 (PMC3709997; doi:10.1371/journal.pone.0069091)

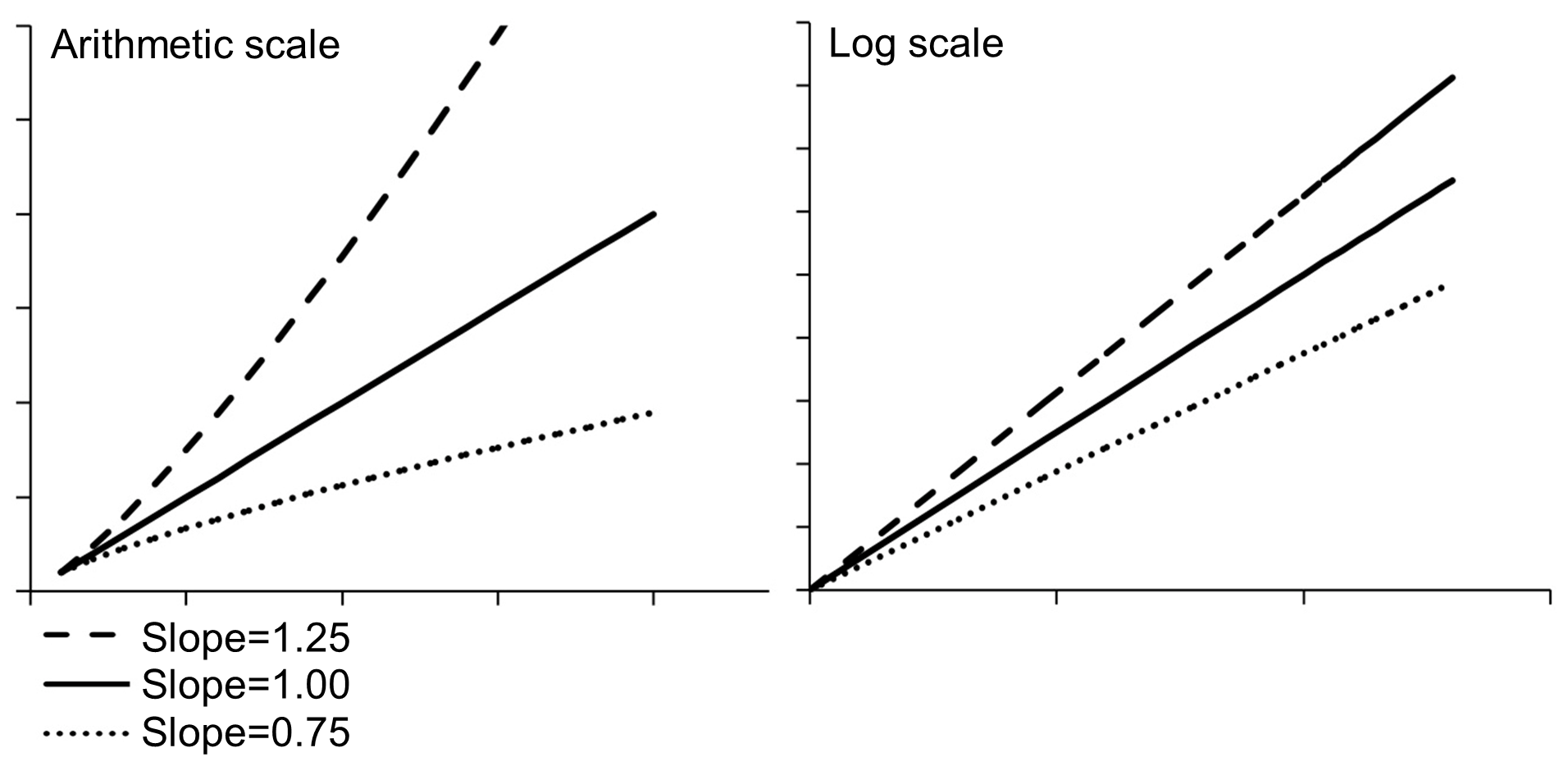

Supplement: Figure S1 — Linear and exponential (increasing at an accelerating rate and increasing at a decelerating rate) relationships in a) untransformed space and b) in log10 transformed space. The slopes for log-transformed lines are indicated. If the x-axis represents population density, the density dependent relationships can be thought of in a similar way to functional responses in predation theory [63]. (TIF) [file pone.0069091.s001.tif]
