# Supplementary material for: Spatial Variation in Population Structure and Its Relation to Movement and the Potential for Dispersal in a Model Intertidal Invertebrate
Source: PLoS One. 2013 Jul 12;8(7):e69091. doi: 10.1371/journal.pone.0069091 (PMC3709997; doi:10.1371/journal.pone.0069091)
Supplement: Table S1 — ANOVA results for densities (ind. m-3, log10[datum+1]-transformed) of swimming Corophium volutator in the upper Bay of Fundy, summer 2010. All sources of variation are random. The degrees of freedom are the same as in Table 2. (DOCX) [file pone.0069091.s002.docx]

Table S1

| Dependent variable | Source of variation | MS | F | p | Variance component | % of Variation |
| --- | --- | --- | --- | --- | --- | --- |
| Juveniles <1.5 mm | Round | 11.668 | 6.4 | 0.007 | 0.125 | 19.8 |
|  | Site | 8.909 | 7.9 | <0.001 | 0.292 | 46.3 |
|  | Round x Site | 1.125 | 6.0 | <0.001 | 0.105 | 16.7 |
|  | Night(Round) | 0.721 | 3.8 | 0.003 | 0.020 | 3.2 |
|  | Night(Round) x Site | 0.189 | 5.1 | <0.001 | 0.051 | 8.1 |
|  | Error | 0.037 |  |  | 0.037 | 5.9 |
| Juveniles 1.5-2.5 mm | Round | 3.327 | 2.3 | 0.133 | 0.024 | 7.3 |
|  | Site | 4.663 | 6.5 | 0.001 | 0.148 | 44.2 |
|  | Round x Site | 0.716 | 5.1 | <0.001 | 0.065 | 19.3 |
|  | Night(Round) | 0.798 | 5.7 | <0.001 | 0.025 | 7.4 |
|  | Night(Round) x Site | 0.140 | 3.6 | <0.001 | 0.034 | 10.2 |
|  | Error | 0.039 |  |  | 0.039 | 11.6 |
| Juveniles 2.5-4 mm | Round | 0.391 | 0.6 | 0.649 | 0.000 | 0.0 |
|  | Site | 3.495 | 7.2 | <0.001 | 0.113 | 47.7 |
|  | Round x Site | 0.485 | 3.8 | <0.001 | 0.040 | 17.0 |
|  | Night(Round) | 0.444 | 3.5 | 0.006 | 0.012 | 5.0 |
|  | Night(Round) x Site | 0.128 | 3.0 | <0.001 | 0.029 | 12.1 |
|  | Error | 0.043 |  |  | 0.043 | 18.2 |
| Adults 4-6 mm | Round | 0.473 | 0.9 | 0.468 | 0.000 | 0.0 |
|  | Site | 2.622 | 8.7 | <0.001 | 0.087 | 48.0 |
|  | Round x Site | 0.301 | 2.2 | 0.020 | 0.018 | 10.1 |
|  | Night(Round) | 0.385 | 2.8 | 0.022 | 0.009 | 5.1 |
|  | Night(Round) x Site | 0.139 | 4.6 | <0.001 | 0.037 | 20.3 |
|  | Error | 0.030 |  |  | 0.030 | 16.6 |
| Adults >6 mm | Round | 0.298 | 2.0 | 0.169 | 0.002 | 4.7 |
|  | Site | 0.437 | 3.0 | 0.030 | 0.011 | 26.7 |
|  | Round x Site | 0.147 | 9.8 | <0.001 | 0.015 | 36.5 |
|  | Night(Round) | 0.012 | 0.8 | 0.575 | 0.000 | 0.0 |
|  | Night(Round) x Site | 0.015 | 1.3 | 0.155 | 0.001 | 2.5 |
|  | Error | 0.012 |  |  | 0.012 | 29.5 |
| Males (>4 mm) | Round | 0.192 | 0.6 | 0.613 | 0.002 | 1.9 |
|  | Site | 1.208 | 6.8 | 0.001 | 0.045 | 36.3 |
|  | Round x Site | 0.179 | 2.5 | 0.008 | 0.020 | 16.1 |
|  | Night(Round) | 0.246 | 3.4 | 0.007 | 0.009 | 7.4 |
|  | Night(Round) x Site | 0.073 | 3.2 | <0.001 | 0.024 | 19.7 |
|  | Error | 0.023 |  |  | 0.023 | 18.7 |
| Non-ovigerous females (>4 mm) | Round | 0.577 | 1.4 | 0.262 | 0.007 | 4.2 |
|  | Site | 1.932 | 7.6 | <0.001 | 0.072 | 41.9 |
|  | Round x Site | 0.253 | 2.6 | 0.006 | 0.028 | 16.4 |
|  | Night(Round) | 0.214 | 2.2 | 0.060 | 0.008 | 4.6 |
|  | Night(Round) x Site | 0.097 | 4.1 | <0.001 | 0.032 | 19.0 |
|  | Error | 0.024 |  |  | 0.024 | 13.8 |
| Ovigerous females | Round | 0.083 | 1.2 | 0.309 | 0.000 | 1.8 |
|  | Site | 0.085 | 1.4 | 0.272 | 0.001 | 7.1 |
|  | Round x Site | 0.061 | 7.6 | <0.001 | 0.006 | 47.4 |
|  | Night(Round) | 0.012 | 1.5 | 0.198 | 0.000 | 1.2 |
|  | Night(Round) x Site | 0.008 | 2.0 | 0.001 | 0.001 | 10.7 |
|  | Error | 0.004 |  |  | 0.004 | 31.8 |
