# Supplementary material for: Spatial Variation in Population Structure and Its Relation to Movement and the Potential for Dispersal in a Model Intertidal Invertebrate
Source: PLoS One. 2013 Jul 12;8(7):e69091. doi: 10.1371/journal.pone.0069091 (PMC3709997; doi:10.1371/journal.pone.0069091)
Supplement: Table S2 — Results for correlation analyses between standardized swimming activity and mud resident variables of Corophium volutator. Significant results are bolded. Patchiness was calculated as variance/mean for the given variable. Body lengths are in mm. (DOCX) [file pone.0069091.s003.docx]

Table S2

| Swimming activity | Resident variable | | df | Correlation | p |
| --- | --- | --- | --- | --- | --- |
| Juveniles <1.5 | | Adult density | 23 | -0.10 | 0.64 |
| Juveniles 1.5-2.5 | | Adult density | 23 | -0.34 | 0.11 |
| Juveniles 2.5-4 | | Adult density | 23 | -0.45 | **0.03** |
| All males | | All female density | 22 | -0.35 | 0.11 |
| All males | | Non-ovig. female density | 22 | -0.33 | 0.14 |
| All males | | Ovigerous female density | 22 | -0.31 | 0.16 |
| All males | | Patchiness non-ovig. females | 22 | -0.19 | 0.41 |
| All males | | Patchiness all females | 22 | -0.21 | 0.34 |
| Males >6 | | All female density | 21 | -0.11 | 0.63 |
| Males >6 | | Non-ovig. female density | 21 | -0.11 | 0.64 |
| Males >6 | | Ovigerous female density | 21 | -0.09 | 0.71 |
| Males >6 | | Patchiness non-ovig. females | 21 | 0.27 | 0.23 |
| Males >6 | | Patchiness all females | 21 | 0.24 | 0.29 |
| All females | | All males density | 23 | -0.37 | 0.09 |
| All females | | Males >6 density | 23 | -0.27 | 0.21 |
| All females | | Patchiness all males | 23 | -0.27 | 0.22 |
| All females | | Patchiness males >6 | 22 | -0.21 | 0.35 |
| Non-ovig. females 4-6 | | All males density | 23 | -0.42 | **0.05** |
| Non-ovig. females 4-6 | | Males 4-6 density | 23 | -0.43 | **0.04** |
| Non-ovig. females 4-6 | | Males >6 density | 23 | -0.30 | 0.17 |
| Non-ovig. females 4-6 | | Patchiness all males | 23 | -0.33 | 0.13 |
| Non-ovig. females 4-6 | | Patchiness males 4-6 | 23 | -0.28 | 0.19 |
| Non-ovig. females 4-6 | | Patchiness males >6 | 22 | -0.28 | 0.21 |
| Non-ovig. females >6 | | All males density | 22 | 0.12 | 0.61 |
| Non-ovig. females >6 | | Males >6 density | 22 | 0.22 | 0.32 |
| Non-ovig. females >6 | | Patchiness all males | 22 | 0.10 | 0.65 |
| Non-ovig. females >6 | | Patchiness males >6 | 22 | 0.23 | 0.30 |
| Ovigerous females | | All males density | 21 | -0.17 | 0.47 |
| Ovigerous females | | Males >6 density | 21 | 0.03 | 0.88 |
| Ovigerous females | | Patchiness all males | 21 | -0.002 | 0.99 |
| Ovigerous females | | Patchiness males >6 | 21 | 0.09 | 0.71 |
